# Investigating the Effect of Enzymatically-Derived Blackcurrant Extract on Skin Staphylococci Using an In Vitro Human *Stratum Corneum* Model

**DOI:** 10.3390/pharmaceutics17040487

**Published:** 2025-04-08

**Authors:** Marija Ćorović, Anja Petrov Ivanković, Ana Milivojević, Klaus Pfeffer, Bernhard Homey, Patrick A. M. Jansen, Patrick L. J. M. Zeeuwen, Ellen H. van den Bogaard, Dejan Bezbradica

**Affiliations:** 1 Faculty of Technology and Metallurgy, University of Belgrade, Karnegijeva 4, 11000 Belgrade, Serbia; amilivojevic@tmf.bg.ac.rs (A.M.); dbez@tmf.bg.ac.rs (D.B.); 2 Innovation Center, Faculty of Technology and Metallurgy, Karnegijeva 4, 11000 Belgrade, Serbia; apetrov@tmf.bg.ac.rs; 3 Department of Microbiology, University Hospital Düsseldorf, Medical Faculty, Heinrich-Heine-University Düsseldorf, 40225 Düsseldorf, Germany; klaus.pfeffer@hhu.de; 4 Department of Dermatology, University Hospital Düsseldorf, Medical Faculty, Heinrich-Heine-University Düsseldorf, 40225 Düsseldorf, Germany; bernhard.homey@med.uni-duesseldorf.de; 5 Department of Dermatology, Radboud University Medical Center, 6525 GA Nijmegen, The Netherlands; patrick.jansen@radboudumc.nl (P.A.M.J.); patrick.zeeuwen@radboudumc.nl (P.L.J.M.Z.); ellen.vandenbogaard@radboudumc.nl (E.H.v.d.B.)

**Keywords:** skin prebiotic, blackcurrant extract, *Staphylococcus aureus*, coagulase-negative staphylococci, *stratum corneum* model

## Abstract

**Background/Objectives**: Numerous intrinsic and extrinsic stressors can disrupt the balance of the skin microbiome, leading to the development of various skin diseases. It has been proven that coagulase-negative staphylococci (CoNS) are important commensals for maintaining skin microbiome homeostasis and fighting cutaneous pathogens such as *Staphylococcus aureus* (*S. aureus*). Here, we examined the influence of polyphenol-rich enzymatic blackcurrant extract (EBCE) on pathogenic coagulase-positive *S. aureus* strains and beneficial CoNS, like *Staphylococcus epidermidis* (*S. epidermidis*), to explore its potential for rebalancing the skin microbiota. **Methods**: The polyphenol profile of EBCE was determined by ultra-high-pressure liquid chromatography–tandem mass spectrometry. Microwell plate assays were employed to study the effect of EBCE on five *S. aureus* strains isolated from the skin of atopic dermatitis patients. An in vitro human *stratum corneum* model was used to test its effect on mixed bacterial cultures. **Results**: EBCE inhibited the growth of all tested *S. aureus* strains by 80–100% at the highest tested concentration after 7 h. No microbial growth was observed at the highest tested EBCE concentration using the *stratum corneum* model inoculated with one selected pathogen (*S. aureus* SA-DUS-017) and one commensal laboratory strain (*S. epidermidis* DSM 20044). The lowest tested concentration did not interfere with *S. aureus* growth but strongly stimulated the growth of *S. epidermidis* (~300-fold colony forming unit increase). In addition, low EBCE concentrations strongly stimulated CoNS growth in microbiome samples taken from the armpits of healthy volunteers that were spiked with *S. aureus* SA-DUS-017. **Conclusions**: These preclinical data support further testing of EBCE-enriched topical preparations as potential cutaneous prebiotics in human studies.

## 1. Introduction

Skin is the human interface with the external environment and is colonized by diverse microorganisms [1]. Healthy human skin is inhabited by commensal microorganisms, with a significant share of coagulase-negative staphylococci (CoNS), while at the same time continuously being exposed to a large number of pathogenic microorganisms [2]. *Staphylococcus aureus* (*S. aureus*) belongs to the coagulase-positive staphylococci (CoPS) and is one of the most common opportunistic pathogens of the skin [3]. It is usually not present on healthy skin, but it often colonizes lesional skin of atopic dermatitis (AD) patients and contributes to the inflammation process in this disease [4]. Disturbed balance of skin microbiota, which is reflected by decreased microbiome diversity and depletion of commensal microorganisms, could be a significant obstacle in combating infections caused by *S. aureus* [5,6,7,8]. Numerous cutaneous microorganisms, including commensal CoNS, are producers of molecules capable of inhibiting the growth and colonisation of other (potentially harmful) microorganisms, primarily *S. aureus*, or change their way of behaving, thus able to reverse microbial dysbiosis of the skin and play a significant role in the prevention and/or treatment of associated skin diseases [9]. A number of comprehensive studies proved that increased CoNS abundance correlates with decreased proliferation of skin pathogens such as *S. aureus*, highlighting their importance for the treatment of AD [10,11,12,13]. Having in mind the importance of these interactions of commensal CoNS with *S. aureus* for shaping up the composition of skin microbiota and fighting skin infections, it is not surprising that there is a heightened interest in agents that could selectively stimulate skin commensals and help restore disturbed homeostasis of the skin microbiome.

We have recently shown that enzymatically derived blackcurrant extract (EBCE), rich in different polyphenols, can exhibit a stimulatory effect on the *Staphylococcus epidermidis* (*S. epidermidis*) DSM 20044 strain and inhibit the growth of the *S. aureus* ATCC 25923 and *Cutibacterium acnes* (*C. acnes*) ATCC 11827 strains [14]. In general, there is growing evidence that certain biomolecules, including those from plant extracts, could influence the growth of skin microbiota representatives [15,16,17]. However, most early-phase skin-prebiotic studies are currently being performed in a liquid medium that does not mimic the surface of the human skin. Therefore, ongoing research aims for simpler and more reliable test methods that utilize systems capable of better mimicking in vivo conditions, particularly the skin surface and the nutrient sources that support bacterial growth.

Hereby, we tried to bridge a gap between early screening phases of prospective skin prebiotics in liquid mediums and more complex examinations on 3D skin models and clinical studies by introducing evaluations using a simple and reliable *stratum corneum* model. In this in vitro model, human callus taken from the heels of healthy volunteers serves as a substrate and nutrient source for bacterial growth. Its suitability was confirmed on single cultures of several skin commensals and pathogens, as well as on complete skin microbiomes of healthy volunteers [18].

In this study, we examined the influence of EBCE obtained using previously optimized enzyme-assisted processes on the growth of skin staphylococci. First, we performed a detailed compositional analysis of the extract regarding present polyphenols. We tested the effect of various EBCE concentrations on the growth of 5 different clinical *S. aureus* strains, isolated from the lesional skin of AD patients, in a microwell plate assay to see if the effect of EBCE is strain-specific and to narrow the concentration range for further examinations. We selected one *S. aureus* strain (SA-DUS-017) that was subsequently grown together with the skin commensal *S. epidermidis* (DSM 20044) and skin microbiome samples using the in vitro human *stratum corneum* model. Selective mannitol salt agar (MSA) plates were used in order to individually monitor the growth of beneficial CoNS and pathogenic CoPS. The data obtained in this study suggest that EBCE may serve as a prebiotic for CoNS and thereby potentially can restore a dysbiotic AD skin microbiome.

## 2. Materials and Methods

### 2.1. Materials

Blackcurrants used for extract preparation were purchased from Drenovac d.o.o., Arilje, Serbia. Viscozyme^®^ L was supplied by Novozymes (Bagsvaerd, Denmark), while Rohapect^®^ MC was a kind donation from AB Enzymes (Darmstadt, Germany). Five clinical isolates of *S. aureus* strains (SA-DUS-number) were collected from lesional skin of patients with AD [19]. Bacterial strain *Staphylococcus epidermidis* DSM 20044 was obtained from Leibniz Institute DSMZ (German Collection of Microorganisms and Cell Culture GmbH, Braunschweig, Germany). Phosphate-buffered saline (PBS) was obtained from Fresenius Kabi GmbH (Graz, Austria). Agar and Columbia blood agar were purchased from Becton, Dickinson and Co., (Sparks, MD, USA). Brain heart infusion (BHI) medium was from Mediaproducts BV (Groningen, The Netherlands), while Muller Hinton (MH) broth was obtained from Sigma-Aldrich (Schnelldorf, Germany). For mannitol salt agar preparation, yeast extract, D-mannitol, NaCl, phenol red, and agar, all purchased from Sigma-Aldrich (Schnelldorf, Germany), were used. All chemicals used for the compositional analysis of blackcurrant extract, as well as chemicals used for extraction buffer preparation, were also supplied from Sigma-Aldrich (Schnelldorf, Germany).

### 2.2. Methods

#### 2.2.1. Preparation of Blackcurrant Extract

Blackcurrant extract was obtained under previously optimized conditions [14]. Briefly, milled blackcurrants and 0.1 M phosphate buffer pH = 4.5 were mixed together in a ratio of 1:10 and incubated for 1 h at 50 °C and 200 rpm with 0.05 mL of Viscozyme^®^ L and Rohapect^®^ (ratio 2:1) per gram of blackcurrant dry matter. After finishing the extraction process, the sample was submerged in a boiling water bath in order to inactivate enzymes and was centrifuged at 6000 rpm for 10 min. Blackcurrant extract was obtained by decanting supernatant and reducing its volume using vacuum evaporation to one-third of the original. The extract was kept frozen at −20 °C prior to use.

#### 2.2.2. Polyphenol Profiling

Ultra-high-pressure liquid chromatography–tandem mass spectrometry (UHPLC–MS/MS) was employed in order to determine the polyphenol profile in EBCE. The experimental work was carried out using an UHPLC 1290 Infinity II instrument (Agilent Technologies, Santa Clara, CA, USA), with a quaternary pump, a column oven, and an autosampler, interfaced to the triple quadrupole mass spectrometer (TQ MS) (Series 6470 TQ, Agilent Technologies, Santa Clara, CA, USA) equipped with Agilent Jet Stream (AJS) electrospray ion source (ESI) source. The separation of compounds was performed using a Zorbax Eclipse Plus C18 column RRHD (50 mm × 2.1 mm; 1.8 μm, Agilent Technologies, Santa Clara, CA, USA). The mobile phase was composed of 0.1% formic acid in water (solvent A) and 0.1% formic acid in methanol (solvent B). The following gradient was used: 0–1 min, 5% B; 1–6 min, 5–50% B; 6–10 min, 50–90% B; 10–12 min, 90% B; 12–14 min, 90–5% B; 14–17 min, 5% B. During analysis, the mobile phase flow rate was 0.30 mL/min, the column temperature was 30 °C, and the injection volume was 2 μL. After separation, the compounds were analyzed using a mass detector. Positive and negative ion modes were recorded (separately), and the instrument was operated in Dynamic Multiple Reaction Monitoring (MRM) mode (to increase the analysis specificity) under following conditions: capillary voltage, 3000 V, nozzle voltage, 1500 V, desolvation gas (nitrogen) temperature, 250 °C, desolvation gas (nitrogen) flow, 12 L/min, nebulizer, 30 psi, sheath gas (nitrogen) temperature, 300 °C, sheath gas (nitrogen) flow, 11 L/min. Different mass spectrometric parameters, such as ionisation mode, fragmentor voltage (FV), and collision energy (CE), were determined for each MRM transition that was monitored. System operation (data collection and processing) was controlled by Agilent Technologies (Santa Clara, CA, USA) MassHunter software (revisions B.06.01 and B.07.00). External calibration curves using a least-squares linear regression analysis were used for the assay of compounds under investigation. Standard stock solutions were prepared in methanol and further diluted with water/0.1% formic acid to obtain calibration standards at concentrations in the range between 0.01 and 2.50 μg/mL. The correlation coefficients of the calibration curve ranged between 0.9936 and 0.9999.

#### 2.2.3. Culturing Bacteria

Bacterial strains were inoculated on Columbia blood agar at 37 °C overnight (o/n). A single colony of each plate was picked and cultured o/n in MH or BHI medium at 37 °C and 225 rpm. For the 96-well plate assay, the o/n culture in the MH medium was diluted 1000 times. 100 µL aliquots of bacterial suspensions were combined with predefined amounts of EBCE diluted in MH medium, reaching a total volume of 200 µL per well. Plates were incubated at 37 °C for 7 h. In the case of the in vitro human *stratum corneum* model, the o/n culture in the BHI medium was diluted 10 times and allowed to grow for another 3 h to reach exponential bacterial growth. The bacteria were collected by centrifugation at 5000 rpm for 5 min, washed twice with PBS, and finally resuspended in 3 mL of PBS. The bacteria were diluted in PBS to a concentration of ~5 × 10^5^ colony forming units (CFU)/mL. Bacterial suspension aliquots of 20 μL for each bacteria were added to each well of the *stratum corneum* model in a 24-well plate, resulting in bacterial concentrations of ~10^4^ CFU of each bacteria per each well. When samples containing human microbiome were examined on the model, approximately the same total number of CFU was used. The bacteria on the model were incubated for 24 h at 32 °C.

#### 2.2.4. Preparation of the Callus-Based Stratum Corneum Model

Human callus powder collected from the heels of 5 healthy volunteers by a callus rasp (Ped Egg™) was mixed, frozen in liquid nitrogen, and subsequently ground up using a Micro Dis-membrator U (B. Braun Biotech International, Melsungen, Germany). The pulverized callus was resuspended in PBS to form a 2% suspension. This suspension was autoclaved and stored at 4 °C until use. For the *stratum corneum* model preparation, a 2% agar solution in PBS was prepared and autoclaved. 1 mL of sterile agar was added to each well of the 24-well plates and, after drying, covered with 100 μL of the previously prepared sterile 2% callus suspension. These plates were dried for 4 h in a sterile hood on a heat block at 42 °C with the lid open and stored at 4 °C prior to use.

#### 2.2.5. Microbial Growth Monitoring

For the microwell plate assay, microbial growth was monitored by the measurements of optical density at 600 nm (OD600). Results were expressed as percentage inhibition/stimulation compared to the control sample (without EBCE). In the case of the *stratum corneum* model, the entire model consisting of agar, callus, and bacteria was removed from the well and placed in a 50 mL tube with 10 mL PBS and then vortexed at high speed for 1 min in order to detach bacteria and suspend them. One ml of the solution containing bacterial cells was transferred to an Eppendorf tube and serially diluted in 10-fold steps. Ten μL of each dilution was placed on MSA plates and incubated at 37 °C for 48 h. Visible yellow and pink colonies originating from CoPS (*S. aureus* SA-DUS-017) and CoNS (*S. epidermidis* DSM 20044 or CoNS from the skin of healthy volunteers), respectively, were counted for each dilution and used for calculating their ratio.

#### 2.2.6. Collection of Microbiome Samples

Microbiome samples from the armpits of 7 healthy volunteers (5 female and 2 male) were collected by swabbing their skin with sample collection swabs (Epicentre Biotechnologies, Madison, WI, USA) in accordance with the previously described procedure [20]. All swabs were transferred to one tube with 10 mL of PBS, centrifuged at 5000 rpm for 5 min, and resuspended in 1 mL of PBS. Twenty μL of this bacterial suspension was placed on the surface of an in vitro *stratum corneum* model and spiked with ~10^3^ CFU *S. aureus* (SA-DUS-017), followed by incubation, extraction, dilution, and growing on MSA plates as described above. The suspension was serially diluted in steps of 10, inoculated twice on Columbia blood agar plates, and incubated at 37 °C for 24 h under both aerobic and anaerobic conditions to determine the initial number of viable colonies in the swabs. For testing the presence of CoPS in swabs, the same serially diluted samples were plated on MSA, and no CoPS were detected (no yellow colonies).

#### 2.2.7. Statistical Analysis

All experiments were performed in duplicates, with the exception of experiments involving microbiome samples, which were conducted in triplicates, and values presented in graphs represent the mean values of independent experiments with error bars showing standard deviation. For the results shown in figures statistical analysis was performed using one-way ANOVA with Dunnett correction for multiple testing by comparing different dosages of EBCE with those of its own control. *p*-values are shown with an asterisk (* *p* < 0.05, ** *p* < 0.01, or *** *p* < 0.001). Statistical analysis was performed using GraphPad Prism 10.4.1.

## 3. Results and Discussion

### 3.1. Determination of the Polyphenolic Profile of Enzymatically Derived Blackcurrant Extract

We previously reported that enzyme-aided extraction of biomolecules from blackcurrant is a suitable way of producing extracts with high polyphenol content and enhanced antioxidant activity and proved presence of four main anthocyanin compounds (delphinidin-3-glucoside, delphinidin-3-rutinoside, cyaniding-3-glucoside, and cyaniding-3-rutinoside) [14,21]. Before testing the effect of EBCE on different skin microbiota representatives, we performed a more detailed analysis of its polyphenol composition. By employing the UHPLC–MS/MS method, we identified 20 more less abundant phenolic compounds (Table 1). This versatile polyphenol profile of EBCE, which includes different important polyphenol classes such as phenolic acids, anthocyanins, flavonols, flavanols, and flavanones, correlates with very high bioactivity (e.g., antioxidant, anticarcinogenic, and photoprotective) previously demonstrated by our research group and other related studies [14,16,22], making it a highly valuable multifunctional ingredient of topical formulations, regardless of its effect on skin microbiota.

### 3.2. Effect of Enzymatic Blackcurrant Extract on Clinical S. aureus Strains

**Figure 1:**
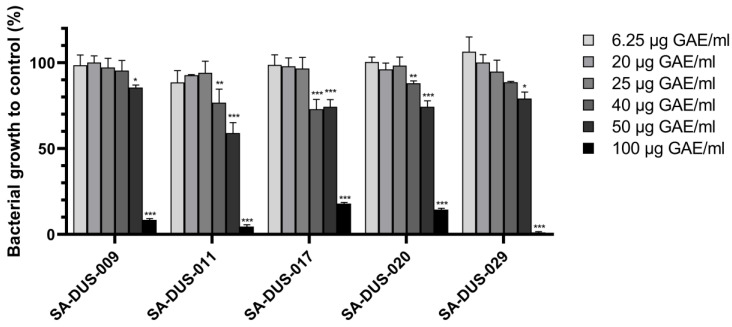
Effect of different EBCE concentrations on the growth of clinical *S. aureus* strains. Input was ~10^4^ CFU for each *S. aureus* SA-DUS strain, and bacterial growth was calculated from OD600 values compared to the control sample of each strain without EBCE supplementation (0 µg GAE/mL). A strong reduction growth is seen at the highest EBCE concentration (100 µg GAE/mL). No *S. aureus* growth-promoting effect was observed for any of the strains. Figures show mean values of two independent experiments with error bars representing standard deviations. *p*-values are shown with an asterisk (* *p* < 0.05, ** *p* < 0.01, or *** *p* < 0.001) for comparing different dosages of EBCE with those of its own control.

By using *S. aureus* ATCC 25923, *S. epidermidis* DSM 20044, and *C. acnes* ATCC 11827 strains grown in a liquid medium enriched with different concentrations of EBCE, we previously demonstrated that it could exhibit a prebiotic-like effect on these particular strains [14]. Knowing that immune-modulatory and protective effects, as well as pathogenicity of skin microbes, are highly strain-specific, the first aim was to investigate the influence of EBCE on five different clinical *S. aureus* strains isolated from the skin of AD patients. The results from microwell plate experiments indicated that at the highest EBCE concentration of 100 gallic acid equivalent (GAE)/mL, bacterial growth was reduced by 80–100% after 7 h of cultivation (Figure 1). In contrast, lower EBCE concentrations had less to no inhibitory effect on the growth of the five tested strains but also no growth-promoting effect. Within the same concentration range, the growth of the commensal CoNS representative, *S. epidermidis* DSM 20044, was stimulated up to ~30% at a concentration of 25 µg GAE/mL and lower, with its growth rate gradually decreasing as concentrations increased [14]. The obtained results align with previous findings, indicating that polyphenols can have both antimicrobial and stimulatory effects on various microbial species, including those associated with both human health and disease [23,24,25,26]. These previous studies corroborate the conclusion that at high doses, EBCE could be used as an antimicrobial agent against *S. aureus* strains, while lower concentrations have the potential to be used as a microbiome-friendly skin care ingredient or skin prebiotic that can stimulate the growth of skin commensals. To test this hypothesis, we conducted experiments with S. *aureus* SA-DUS-017 as a model strain and the previously used *S. epidermidis* DSM 20044 commensal strain by growing them together on an in vitro human *stratum corneum* model.

### 3.3. Effect of Enzymatic Blackcurrant Extract on the Growth of S. aureus SA-DUS-017 and S. epidermidis DSM 20044 Co-Cultured on the Stratum Corneum Model

**Figure 2:**
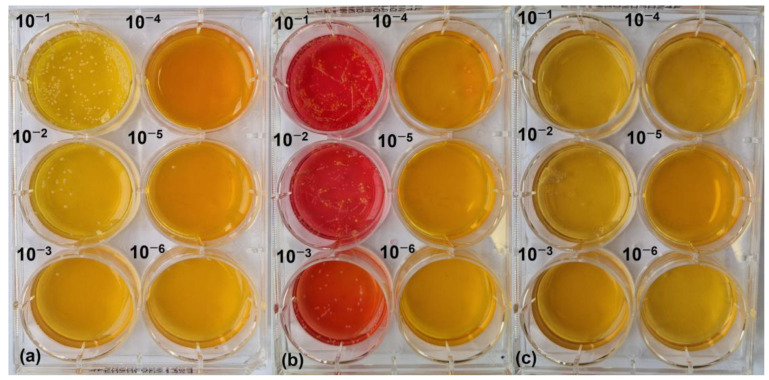
Representative photographs of CoNS (pink) and CoPS (yellow) colonies in a serially diluted (**a**) control sample without EBCE, (**b**) a sample supplemented with 3.8 µg GAE/cm^2^ of EBCE, and (**c**) a sample supplemented with 76.0 µg GAE/cm^2^ of EBCE. These pictures illustrate respectively how *S. aureus* AD-DUS-017 is outgrowing *S. epidermidis* DSM 20044 in the control sample, how *S. epidermidis* DSM 20044 is outgrowing *S. aureus* SA-DUS-017 in a sample with the low EBCE concentration, and how both strains are completely inhibited in the sample with the high EBCE concentration. Samples represent microbial growth of viable colonies extracted from the *stratum corneum* model that was inoculated with ~10^4^ CFU of both strains.

We tested EBCE in a more complex environment, more similar to in vivo conditions, to examine how it affects the growth of the skin commensal *S. epidermidis* DSM 20044 and the skin pathogen *S. aureus* SA-DUS-017. Therefore, both bacterial strains were inoculated on the *stratum corneum* model, and microbial growth with and without EBCE supplementation was compared. We found that the CoPS representative (*S. aureus*) is outgrowing the CoNS (*S. epidermidis*) representative in the control sample (Figure 2a), that the CoNS representative is outgrowing the CoPS representative in a sample with 3.8 µg GAE/cm^2^ of EBCE (Figure 2b), and that no viable colonies of both strains are present in a sample with 76.0 µg GAE/cm^2^ of EBCE (Figure 2c).

**Figure 3:**
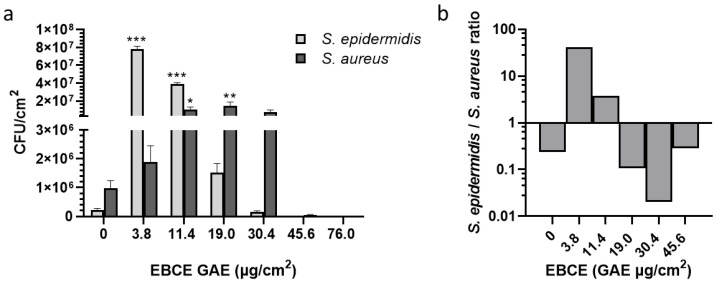
Effect of different EBCE concentrations on the growth of *S. aureus* SA-DUS-017 and *S. epidermidis* DSM 20044 grown together on the in vitro *stratum corneum* model. Results represent microbial growth of viable colonies extracted from the *stratum corneum* model inoculated with ~10^4^ CFU of both strains. (**a**) the CFU values of both individual strains, and (**b**) the *S. epidermidis*/*S. aureus* ratio calculated from the mean CFU values of the individual strains (log-scale). Figures show the mean values of two independent experiments with error bars representing standard deviations. *p*-values are shown with an asterisk: * *p* < 0.05, ** *p* < 0.01, or *** *p* < 0.001.

As shown in Figure 3a, the lowest tested EBCE concentration, 3.8 µg GAE/cm^2^, did exhibit a mild increase (but not statistically significant) of growth on *S. aureus* SA-DUS-017 compared to the control (no EBCE), while the growth of *S. epidermidis* was strongly stimulated. This growth stimulation of *S. epidermidis*, however, decreases at higher EBCE concentrations. In contrast, between 11.4 µg GAE/cm^2^ and 30.4 µg GAE/cm^2^ of EBCE, a stimulation of *S. aureus* growth was observed. Similarly, as in the microwell plate experiment, *S. aureus* AS-DUS-017 growth was strongly inhibited by high EBCE concentrations (45.6–76.0 µg GAE/cm^2^). These concentrations also demonstrated a strong inhibitory effect on the *S. epidermidis* strain, implicating that EBCE at high concentrations does not show selectivity. Based on these results, it is obvious that only the lowest tested EBCE concentration, 3.8 µg GAE/cm^2^, exhibits a significant prebiotic-like effect leading to ~300-fold increase in CFU of *S. epidermidis* compared to the control without EBCE. Having in mind that *S. epidermidis* has been recently suggested as a skin probiotic due to its role in collagen type I induction, skin ceramide level increase, prevention of water loss of damaged skin, and generally improving the skin’s protective function [27,28,29], this is a noteworthy result. *S. epidermidis*/*S. aureus* ratio at the lowest tested concentration was 41.2, which is significantly higher compared to the control sample without EBCE (ratio of 0.23) in which the *S. aureus* strain overgrew the CoNS representative (Figure 3b). Compared to our previous study, where the effect of EBCE on the same *S. epidermidis* strain was tested in a liquid culture, the extract showed a significantly higher degree of stimulation on the callus-based *stratum corneum* model, which highlights the differences in results obtained across different systems [14]. In addition, an optimum concentration change toward lower EBCE concentrations was observed, compared to experiments in a liquid medium, which could be mostly ascribed to differences in nutrients available for bacterial growth. While media used for the cultivation of microorganisms are generally rich sources of nutrients that provide high growth rates and a low susceptibility to other supplemented agents, the *stratum corneum* model mimics the conditions of human skin by providing proteins from dead corneocytes as the main nutrient source. Similar discrepancies in the effect of several cosmetic ingredients on the growth and virulence factor expression in the *S. aureus* C-29 strain were previously reported in a multicomponent keratin-based in vitro model compared with the BHI growth medium [23]. It is clear that after the preliminary screening of potential topical agents targeting the skin microbiota in liquid media, using a *stratum corneum* model for medium-throughput testing is appropriate before progressing to more complex studies with organotypic 3D skin models and in vivo trials.

### 3.4. Effect of Enzymatic Blackcurrant Extract on the Growth of S. aureus SA-DUS-017 and Skin Microbiome Samples Co-Cultured on the Stratum Corneum Model

**Figure 4:**
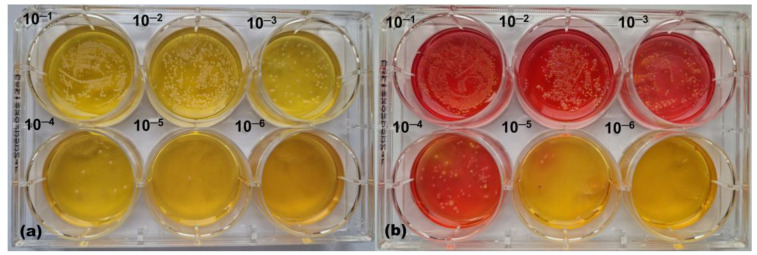
Representative photographs of CoNS (pink) and CoPS (yellow) colonies in (**a**) a serially diluted control sample without EBCE and (**b**) a sample supplemented with 3.8 µg GAE/cm^2^ of EBCE. These pictures illustrate how *S. aureus* SA-DUS-017 is outgrowing CoNS in the control sample, and CoNS are outgrowing *S. aureus* SA-DUS-017 in the sample with EBCE. Samples represent the growth of viable colonies extracted from the *stratum corneum* model inoculated with ~10^3^ CFU of *S. aureus* SA-DUS-017 and ~10^4^ CFU of aerobic bacteria from the armpits of seven healthy volunteers.

After demonstrating the prebiotic potential of EBCE on the callus-based *stratum corneum* model with two bacterial strains, we tested its effect on skin microbiome samples taken from the armpits of healthy volunteers spiked with *S. aureus* SA-DUS-017. As proven by plating microbiome samples on MSA plates, CoPS were not present in armpit swabs, indicating that all detected yellow colonies in these samples originated from *S. aureus* SA-DUS-017 growth. In the absence of EBCE, *S. aureus* SA-DUS-017 outgrew armpit CoNS on the *stratum corneum* model (Figure 4a). In contrast, CoNS from the armpit microbiota outgrew *S. aureus* SA-DUS-017 in the same system when supplemented with EBCE at a concentration of 3.8 µg GAE/cm^2^ (Figure 4b).

**Figure 5:**
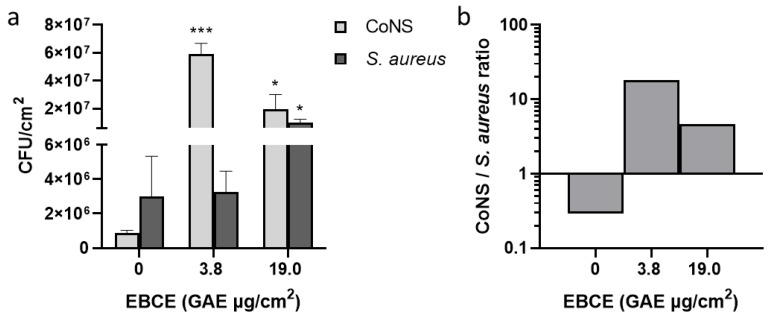
The effect of two different EBCE concentrations on the growth of *S. aureus* SA-DUS-017 and CoNS bacteria from the skin of healthy volunteers grown together for 24 h on the in vitro *stratum corneum* model. Results are representing microbial growth of viable colonies extracted from the *stratum corneum* model inoculated with ~10^3^ CFU of *S. aureus* SA-DUS-017 and ~10^4^ CFU of aerobic bacteria from the armpits of seven healthy volunteers. (**a**) CFU of CoNS and *S. aureus* SA-DUS-017 and (**b**) CoNS/*S. aureus* SA-DUS-017 ratio calculated from mean values of CFU number. Figures are showing mean values of two independent experiments with error bars, which represent standard deviations. *p*-values are shown with an asterisk: * *p* < 0.05 or *** *p* < 0.001.

After 24 h of growth on the *stratum corneum* model without EBCE supplementation (control), an approximately 3-fold higher CFU number of *S. aureus* SA-DUS-017 was detected compared to total CoNS, corresponding to a ratio of 0.29 (Figure 5). On the other hand, both EBCE concentrations (3.8 µg GAE/cm^2^ and 19.0 µg GAE/cm^2^) showed a positive effect on the CoNS/*S. aureus* SA-DUS-017 ratio but to a different extent. While a concentration of 19.0 µg GAE/ stimulated both CoNS and *S. aureus* SA-DUS-017, resulting in a ratio of 1.95, a dose of 3.8 µg GAE/cm^2^ led to a significant stimulation of CoNS but not *S. aureus* SA-DUS-017, resulting in a CoNS/*S. aureus* SA-DUS-017 ratio of 18.2 after 24 h of growth on the *stratum corneum* model. It should be emphasized that lower EBCE concentrations, which exhibited a stimulatory effect on CoNS, were not capable of inhibiting *S. aureus* SA-DUS-017 growth (see Figure 1). However, the stimulatory effect that EBCE showed on CoNS could potentially indirectly lead to a reduced growth of *S. aureus*. Recently, it was shown that several CoNS can significantly contribute to skin homeostasis by preventing colonization of pathogens, including *S. aureus*, through various mechanisms [11,12,30]. For example, the most abundant CoNS representative, *S. epidermidis*, could compete with *S. aureus* for nutrients and adhesion sites on the skin surface and prevent *S. aureus* biofilm formation by serine protease (Esp) activity [31]. It releases bacteriocins and other antimicrobial molecules that can cause *S. aureus* cell lysis. In addition, it can activate TRL2 receptors that are able to subsequently induce the production of human defensins hBD2 and hBD3 by keratinocytes [32,33]. Furthermore, EBCE can be applied in prebiotic products intended for topical application, during and/or after antibiotic treatments, to fight potential skin microbiome dysbiosis by supporting the repopulation of the skin with commensal CoNS. Further studies should be done to confirm the effectiveness of increased CoNS growth by EBCE on *S. aureus* proliferation and virulence factors formation. Also, topical formulations with incorporated EBCE need to be tested in in vitro organotypic 3D skin models and on healthy volunteers and AD patients to prove its in vivo efficacy in rebalancing the human skin microbiome.

## 4. Conclusions

The specific mechanism by which individual commensals, including CoNS, cooperate with the skin’s immune system and keratinocytes to combat pathogen colonization is not fully understood. Nevertheless, it is clear that the diversity and abundance of bacteria present on the skin are important factors in maintaining skin health. Results obtained within the current study demonstrate that enzymatically derived blackcurrant extract is a potent agent capable of selectively stimulating the in vitro growth of commensal CoNS on the human *stratum corneum* model. This serves as a firm basis for further investigation in more complex systems like in vitro 3D skin equivalents or in vivo clinical trials, which will shed further light on the effect of EBCE on the cutaneous microbiome.

## Figures and Tables

**Table 1:** Polyphenolic profile of enzymatically derived blackcurrant extract.

Compound	Retention Time (min)	Ionisation Mode	Transition	FV (V)	CE (V)	Concentration in EBCE (µg/mL)
Gallic acid	1.65	Negative	169 ⟶ 125	100	10	2.22 ± 0.02
Protocatechuic acid	3.38	Negative	153 ⟶ 109	100	9	1.58 ± 0.03
*p*-Hydroxybenzoic acid	4.80	Negative	137 ⟶ 93	100	10	1.17 ± 0.02
Catechin	5.05	Negative	289 ⟶ 245	100	10	0.32 ± 0.02
Caffeic acid	5.32	Negative	179 ⟶ 135	100	10	1.71 ± 0.05
Vanillic acid	5.50	Negative	167 ⟶ 108	100	15	0.11 ± 0.06
Syringic acid	5.92	Negative	197 ⟶ 182	100	12	<0.01
Epicatechin	6.00	Negative	289 ⟶ 245	104	10	0.10 ± 0.02
Malvidin-3-glucoside	6.20	Positive	493.1 ⟶ 331.1	116	24	1.62 ± 0.46
*p*-Coumaric acid	6.60	Negative	163 ⟶ 119	100	9	7.27 ± 0.51
Taxifolin	6.82	Negative	302.7 ⟶ 284.8	100	14	0.26 ± 0.03
*t*-Ferulic acid	6.90	Negative	193 ⟶ 134	100	11	0.59 ± 0.06
Salicylic acid	7.40	Negative	137 ⟶ 93	100	10	0.11± 0.08
Resveratrol	7.41	Negative	227 ⟶ 185	100	20	0.05 ± 0.03
Rutin	7.70	Negative	609 ⟶ 300	100	42	12.42 ± 0.28
Ellagic acid	7.90	Negative	301 ⟶ 257	100	35	0.07 ± 0.03
Myricetin	8.10	Negative	316.7 ⟶ 150.9	100	26	8.74 ± 1.69
Naringenin	8.74	Negative	271 ⟶ 151	100	16	0.06 ± 0.01
Quercetin	8.80	Negative	301 ⟶ 179	100	15	1.84 ± 0.08
Kaempferol	9.40	Negative	285 ⟶ 93.4	100	52	0.35 ± 0.07

## Data Availability

The original contributions presented in this study are included in the article. Further inquiries can be directed to the corresponding author.

## References

[1] Grice E.A., Segre J.A. (2011). The skin microbiome. Nat. Rev. Microbiol..

[2] Parlet C.P., Brown M.M., Horswill A.R. (2019). Commensal staphylococci influence Staphylococcus aureus skin colonization and disease. Trends Microbiol..

[3] Bier K., Schittek B. (2021). Beneficial effects of coagulase-negative Staphylococci on Staphylococcus aureus skin colonization. Exp. Dermatol..

[4] Kim J., Kim B.E., Ahn K., Leung D.Y. (2019). Interactions between atopic dermatitis and Staphylococcus aureus infection: Clinical implications. Allergy Asthma Immunol. Res..

[5] Zhu Y., Yu X., Cheng G. (2023). Human skin bacterial microbiota homeostasis: A delicate balance between health and disease. mLife.

[6] Cogen A., Nizet V., Gallo R. (2008). Skin microbiota: A source of disease or defence?. Br. J. Dermatol..

[7] van der Krieken D.A., Rikken G., Ederveen T.H., Jansen P.A., Rodijk-Olthuis D., Meesters L.D., van Vlijmen-Willems I.M., van Cranenbroek B., van der Molen R.G., Schalkwijk J. (2023). Gram-positive anaerobic cocci guard skin homeostasis by regulating host-defense mechanisms. iScience.

[8] Nakatsuji T., Brinton S.L., Cavagnero K.J., O’Neill A.M., Chen Y., Dokoshi T., Butcher A.M., Osuoji O.C., Shafiq F., Espinoza J.L. (2023). Competition between skin antimicrobial peptides and commensal bacteria in type 2 inflammation enables survival of *S. aureus*. Cell Rep..

[9] Byrd A.L., Belkaid Y., Segre J.A. (2018). The human skin microbiome. Nat. Rev. Microbiol..

[10] Nakatsuji T., Chen T.H., Narala S., Chun K.A., Two A.M., Yun T., Shafiq F., Kotol P.F., Bouslimani A., Melnik A.V. (2017). Antimicrobials from human skin commensal bacteria protect against Staphylococcus aureus and are deficient in atopic dermatitis. Sci. Transl. Med..

[11] Iwase T., Uehara Y., Shinji H., Tajima A., Seo H., Takada K., Agata T., Mizunoe Y. (2010). Staphylococcus epidermidis Esp inhibits Staphylococcus aureus biofilm formation and nasal colonization. Nature.

[12] Paharik A.E., Parlet C.P., Chung N., Todd D.A., Rodriguez E.I., Van Dyke M.J., Cech N.B., Horswill A.R. (2017). Coagulase-negative staphylococcal strain prevents Staphylococcus aureus colonization and skin infection by blocking quorum sensing. Cell Host Microbe.

[13] Chin D., Goncheva M.I., Flannagan R.S., Deecker S.R., Guariglia-Oropeza V., Ensminger A.W., Heinrichs D.E. (2021). Coagulase-negative staphylococci release a purine analog that inhibits Staphylococcus aureus virulence. Nat. Commun..

[14] Petrov Ivanković A., Milivojević A., Ćorović M., Simović M., Banjanac K., Jansen P., Vukoičić A., Van den Bogaard E., Bezbradica D. (2023). In vitro evaluation of enzymatically derived blackcurrant extract as prebiotic cosmetic ingredient: Extraction conditions optimization and effect on cutaneous microbiota representatives. Chem. Biol. Technol. Agric..

[15] D’Arcangelo S., Di Fermo P., Diban F., Ferrone V., D’Ercole S., Di Giulio M., Di Lodovico S. (2024). Staphylococcus aureus/Staphylococcus epidermidis from skin microbiota are balanced by Pomegranate peel extract: An eco-sustainable approach. PLoS ONE.

[16] Petrov Ivanković A., Ćorović M., Milivojević A., Simović M., Banjanac K., Veljković M., Bezbradica D. (2024). Berries pomace valorization: From waste to potent antioxidants and emerging skin prebiotics. Int. J. Fruit Sci..

[17] Ivanovska A., Gajić I.S., Mravik Ž., Reljić M., Ilić-Tomić T., Savić I., Luxbacher T., Lađarević J. (2024). Transforming discarded walnut green husk into a resource of valuable compounds for colored bioactive textiles with a focus on circular economy concept. Dyes Pigm..

[18] van der Krieken D.A., Ederveen T.H., Van Hijum S.A., Jansen P.A., Melchers W.J., Scheepers P.T., Schalkwijk J., Zeeuwen P.L. (2016). An in vitro model for bacterial growth on human stratum corneum. Acta Derm. Venereol..

[19] Fyhrquist N., Muirhead G., Prast-Nielsen S., Jeanmougin M., Olah P., Skoog T., Jules-Clement G., Feld M., Barrientos-Somarribas M., Sinkko H. (2019). Microbe-host interplay in atopic dermatitis and psoriasis. Nat. Commun..

[20] Zeeuwen P.L., Boekhorst J., van den Bogaard E.H., de Koning H.D., van de Kerkhof P.M., Saulnier D.M., van Swam I.I., van Hijum S.A., Kleerebezem M., Schalkwijk J. (2012). Microbiome dynamics of human epidermis following skin barrier disruption. Genome Biol..

[21] Petrov Ivanković A., Ćorović M., Milivojević A., Blagojević S., Radulović A., Pjanović R., Bezbradica D. (2024). Assessment of enzymatically derived blackcurrant extract as cosmetic ingredient—Antioxidant properties determination and in vitro diffusion study. Pharmaceutics.

[22] Ejaz A., Waliat S., Afzaal M., Saeed F., Ahmad A., Din A., Ateeq H., Asghar A., Shah Y.A., Rafi A. (2023). Biological activities, therapeutic potential, and pharmacological aspects of blackcurrants (*Ribes nigrum* L.): A comprehensive review. Food Sci. Nutr..

[23] Uehara Y., Shimamura Y., Takemura C., Suzuki S., Masuda S. (2025). Effects of cosmetic ingredients on growth and virulence factor expression in Staphylococcus aureus: A comparison between culture medium and in vitro skin model medium. AIMS Microbiol..

[24] Alves-Santos A.M., Sugizaki C.S.A., Lima G.C., Naves M.M.V. (2020). Prebiotic effect of dietary polyphenols: A systematic review. J. Funct. Foods.

[25] Manso T., Lores M., de Miguel T. (2021). Antimicrobial activity of polyphenols and natural polyphenolic extracts on clinical isolates. Antibiotics.

[26] Sun M., Deng Y., Cao X., Xiao L., Ding Q., Luo F., Huang P., Gao Y., Liu M., Zhao H. (2022). Effects of natural polyphenols on skin and hair health: A review. Molecules.

[27] Negari I.P., Keshari S., Huang C.-M. (2021). Probiotic activity of Staphylococcus epidermidis induces collagen type I production through FFaR2/p-ERK signaling. Int. J. Mol. Sci..

[28] Zheng Y., Hunt R.L., Villaruz A.E., Fisher E.L., Liu R., Liu Q., Cheung G.Y., Li M., Otto M. (2022). Commensal Staphylococcus epidermidis contributes to skin barrier homeostasis by generating protective ceramides. Cell Host Microbe.

[29] Swaney M.H., Kalan L.R. (2022). Two-for-one: Dual host-microbe functions of S. epidermidis Sph. Cell Host Microbe.

[30] Stacy A., Belkaid Y. (2019). Microbial guardians of skin health. Science.

[31] Vandecandelaere I., Depuydt P., Nelis H.J., Coenye T. (2014). Protease production by Staphylococcus epidermidis and its effect on Staphylococcus aureus biofilms. Pathog. Dis..

[32] Volz T., Kaesler S., Draing C., Hartung T., Röcken M., Skabytska Y., Biedermann T. (2018). Induction of IL-10-balanced immune profiles following exposure to LTA from Staphylococcus epidermidis. Exp. Dermatol..

[33] Lai Y., Cogen A.L., Radek K.A., Park H.J., MacLeod D.T., Leichtle A., Ryan A.F., Di Nardo A., Gallo R.L. (2010). Activation of TLR2 by a small molecule produced by Staphylococcus epidermidis increases antimicrobial defense against bacterial skin infections. J. Investig. Dermatol..

